# Three Bacterial DedA Subfamilies with Distinct Functions and Phylogenetic Distribution

**DOI:** 10.1128/mbio.00028-23

**Published:** 2023-03-01

**Authors:** Horia Todor, Nadia Herrera, Carol A. Gross

**Affiliations:** a Department of Microbiology and Immunology, University of California, San Francisco, San Francisco, California, USA; b Department of Cell and Tissue Biology, University of California, San Francisco, San Francisco, California, USA; c California Institute of Quantitative Biology, University of California, San Francisco, San Francisco, California, USA; California Institute of Technology

**Keywords:** DedA, AlphaFold, antibiotics, microbiology

## Abstract

Recent studies in bacteria have suggested that the broadly conserved but enigmatic DedA proteins function as undecaprenyl-phosphate (UndP) flippases, recycling this essential lipid carrier. To determine whether all DedA proteins have UndP flippase activity, we performed a phylogenetic analysis and correlated our findings to previously published experimental results and predicted structures. We uncovered three major DedA subfamilies: one contains UndP flippases, the second contains putative phospholipid flippases and is associated with aerobic metabolism, and the third is found only in specific Gram-negative phyla.

## OBSERVATION

DedA family proteins are broadly distributed and nearly ubiquitous in eukaryotes, archaea, and bacteria. The structure of the DedA domain has not been solved, but computational (1) and topological (2) approaches indicate similarity to other transporter families (2). Eukaryotic VMP1, TVP38, and TMEM41B DedA proteins are central players in autophagy, while bacterial DedA family members play roles in colistin resistance, cell division, and pH sensitivity (detailed below).

Our understanding of DedA proteins was greatly bolstered by recent studies demonstrating that eukaryotic DedA homologs function as phospholipid scramblases (3–6) and that some bacterial DedA proteins are undecaprenyl phosphate (UndP) flippases (7, 8). UndP is the essential lipid carrier for the biogenesis of peptidoglycan and other bacterial surface polymers and must be recycled from the outer leaflet of the inner membrane to the cytoplasmic side—an essential function not previously associated with a gene.

These observations raise an important question: are all bacterial DedAs UndP flippases? Several factors suggest the answer is no. First, most bacteria have numerous DedAs: Escherichia coli has 8 and Bacillus subtilis has 6. Second, only some DedAs exhibit UndP flippase activity (7). In B. subtilis, deletion of DedA homologs *yngC* and *ykoX* sensitized cells to the UndP-targeting drug MX2401, but deletion of the other 4 DedAs did not (7). In E. coli, at least one DedA protein is required for viability (due to the essentiality of UndP flippase activity), but only 4/8 DedA members are able to support viability as the sole DedA (9). Finally, some bacterial DedAs resemble eukaryotic DedAs, which are phospholipid scramblases (3–6).

To better understand the role(s) of bacterial DedAs, we performed a phylogenetic analysis of the DedA family in bacteria and found three major subfamilies, which largely correspond to the clusters of orthologous genes (COG) families COG0586, COG0398, and COG1238. Our bioinformatic analysis found that each subfamily exhibited a distinct phylogenetic distribution, genomic context, and functional residues, implying related but distinct functions and laying the groundwork for experimental characterization.

### DedA proteins are divided into 3 major subfamilies.

**FIG 1:**
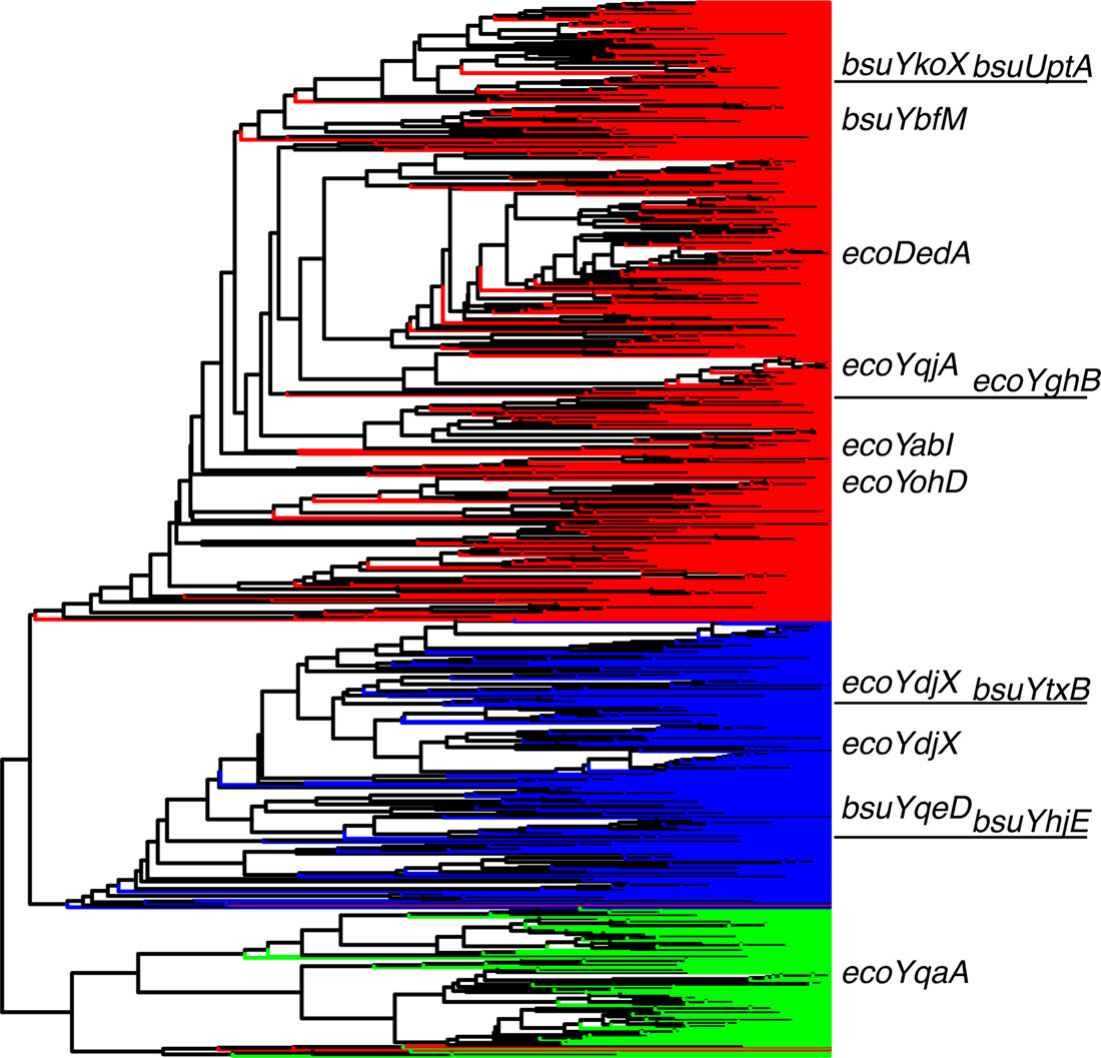
UPGMA tree of 1,000 bacterial DedA family proteins revealed 3 distinct subfamilies, which are congruent with COG0586 (red), COG0398 (blue), and COG1238 (green). All DedAs from E. coli and B. subtilis are indicated.

To determine whether there are distinct DedA subfamilies within bacteria, we first identified all DedA homologs in ~6,000 representative bacterial genomes using the PF09335 (DedA family) motif (see “Methods”) (see Table S1 in the supplemental material). We then used a representative subsample of these sequences to construct a tree (Fig. 1) and found three major subfamilies. These subfamilies corresponded to previously computed clusters COG0586, COG0398, and COG1238, and each contained DedAs from multiple bacterial phyla (Table 1, Fig. 1), suggesting that their divergence predated the last bacterial common ancestor.

10.1128/mbio.00028-23.3 TABLE S1 All of the DedA homologs used in the analysis. Download Table S1, XLSX file, 4.5 MB. Copyright © 2023 Todor et al. 2023 Todor et al. https://creativecommons.org/licenses/by/4.0/ This content is distributed under the terms of the Creative Commons Attribution 4.0 International license.

**FIG 2:**
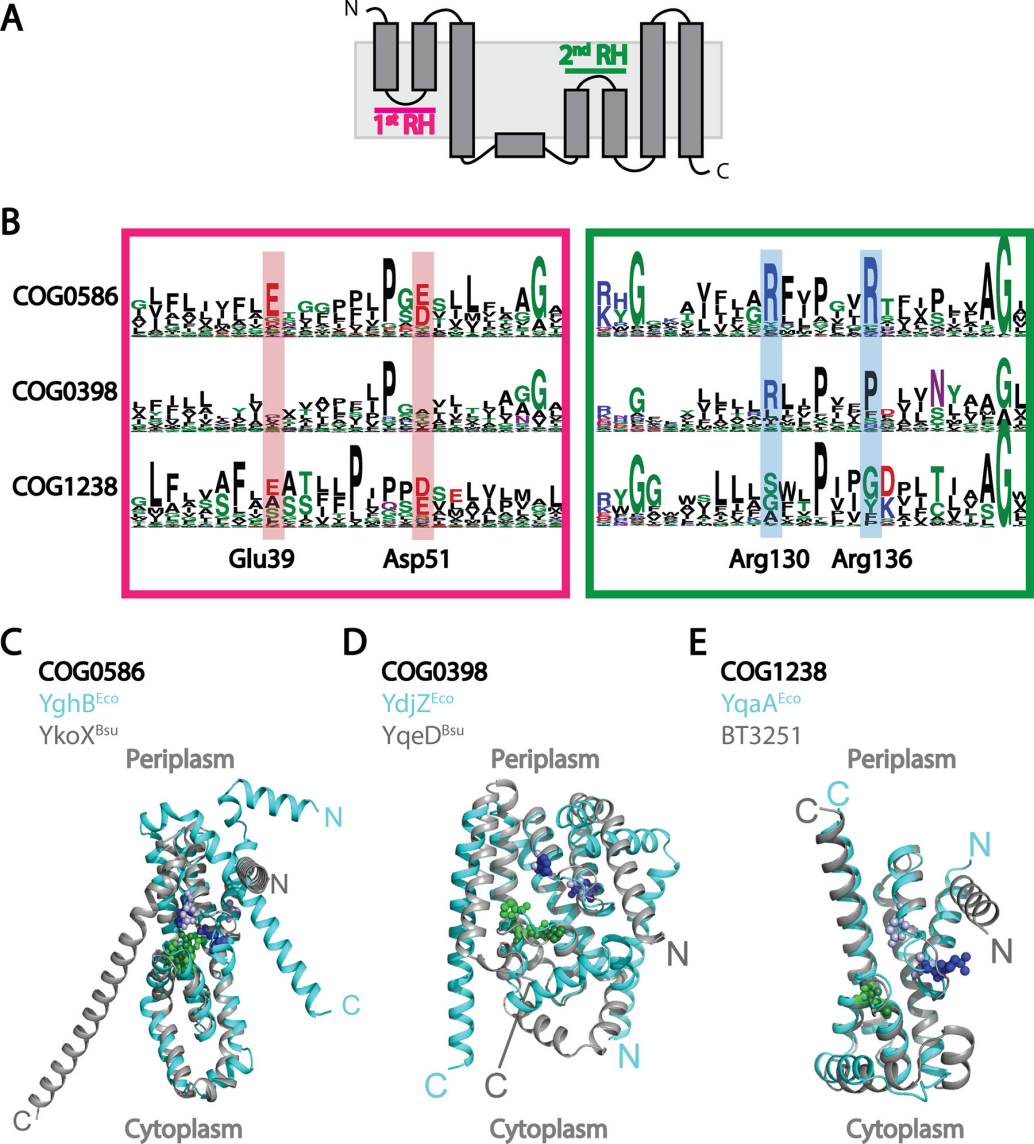
(A) Schematic of core DedA domain topology (2), depicting helices and reentrant helices (RH). (B) Consensus motif of the tips of the first and second reentrant helices in the 3 major DedA subfamilies, with conserved and essential residues highlighted. Residue numbering follows that for YghB^Eco^. (C to E) AlphaFold models of representative DedA proteins in each subfamily, showing differing orientation of the N- and C-terminal helices and conserved structure of the core domain. Periplasmic and cytoplasmic orientations are based on topological study of YqjA^Eco^ (2). Colored spheres represent residues at the tip of the first reentrant helix (dark blue and light purple) and second reentrant helix (green and dark green) for the first and second proteins, respectively. RMSDs for the entire protein alignment and for the core DedA domain were 8.413 Å and 3.899 Å for COG0586, 0.978 Å and 0.828 Å for COG0398, and 0.968 Å and 0.972 Å for COG1238.

We next performed an AlphaFold-based (10) structural analysis to assess differences between families (Fig. S1; Fig. 2C to E). We found that although the structure of the core DedA domain was largely conserved (root mean square deviation [RMSD], 6 to 8 Å), the placement of the N and C termini varied in both location and membrane orientation (cytoplasmic versus periplasmic) (Fig. S1). N- and C-terminal locations and orientations were conserved within subfamilies (Fig. 2C to E), indicating that they are not caused solely by the mishandling of the C-terminal dimerization helices (2) but may reflect functional differences. The structural conservation of the DedA core domain and the variability of N- and C-terminal helices suggested that DedA subfamilies share a basic function (lipid scrambling or flipping) but vary in their substrate.

10.1128/mbio.00028-23.1 FIG S1 Aligned DedA from each of the three families from E. coli: YghB (cyan, COG0586), YdjZ (orange, COG0398), and YqaA (gray, COG1238). Alignment is shown for the DedA domain only (A) or entire protein (B), highlighting the similarity of the core domain and the differences in the orientation of the N- and C-terminal helices. Periplasmic and cytoplasmic orientations are based on topological study of YqjA^Eco^ (2). RMSDs of the DedA core domains were as follows: YghB-YqaA, 8.579 Å; YghB-YdjZ, 6.094 Å. RMSDs of the entire protein were as follows: YghB-YqaA, 8.578 Å; YghB-YdjZ, 6.242 Å. Blue, purple, and light blue spheres show resides at the tip of the first reentrant helix for YghB, YqaA, and YdjZ, respectively. Green, yellow, and dark green spheres show residues at the tip of the second reentrant helix for YghB, YqaA, and YjdZ, respectively. Download FIG S1, TIF file, 6.2 MB. Copyright © 2023 Todor et al. 2023 Todor et al. https://creativecommons.org/licenses/by/4.0/ This content is distributed under the terms of the Creative Commons Attribution 4.0 International license.

### COG0586 DedA proteins are putative UndP flippases.

The COG0586 subfamily accounts for ~58% of bacterial DedAs. It is found in all major bacterial clades except for the peptidoglycan-less *Tenericutes* and the *Thermotogota* (Table 1). Almost all experimentally studied bacterial DedAs are in this family, including the sole (and essential) DedA of Borrelia burgdorferi (11), the target of the antibiotic halicyclamine A in Mycobacterium smegmatis (12), as well as DedAs implicated in colistin resistance through 4-amino-4-deoxy-l-arabinose modification of lipid A in Klebsiella pneumoniae (13), Enterobacter cloacae (14), Burkholderia thailandensis (15), Burkholderia glumae (16), and others (17–19).

**TABLE 1:** The distribution of DedA family proteins in different bacterial clades

Taxonomic group^*a*^	No. of taxonomic group members with indicated no. of DedA proteins, per COG subfamily																				All genomes
	COG0586								COG0398								COG1238				
	0	1	2	3	4	5	6	7+	0	1	2	3	4	5	6	7+	0	1	2	3	
*Gammaproteobacteria*	202	246	150	148	68	118	29	6	542	242	125	43	12	0	3	0	242	575	120	30	967
*Actinobacteria*	12	129	193	136	92	93	67	88	363	371	70	5	1	0	0	0	753	51	6	0	810
*Alpahaproteobacteria*	270	188	116	75	14	3	0	0	311	224	117	11	3	0	0	0	182	363	108	13	666
*Bacilli*	180	140	144	81	55	33	17	11	83	268	147	100	42	17	3	1	620	32	9	0	661
*Clostridia*	264	84	53	28	5	3	3	1	174	134	72	34	18	5	1	3	426	15	0	0	441
*Betaproteobacteria*	18	69	128	46	23	10	8	5	221	73	12	1	0	0	0	0	103	194	9	1	307
*Flavobacteriia*	106	73	4	4	0	0	0	0	163	20	3	1	0	0	0	0	143	44	0	0	187
*Bacteroidia*	25	126	20	0	0	0	0	0	171	0	0	0	0	0	0	0	28	137	6	0	171
*Cyanobacteria*	17	72	50	15	1	0	0	0	12	32	70	29	10	1	1	0	152	3	0	0	155
*Deltaproteobacteria*	35	37	27	13	4	2	0	0	26	30	31	25	5	1	0	0	58	48	12	0	118
*Epsilonproteobacteria*	1	0	22	58	7	1	1	0	77	5	8	0	0	0	0	0	50	40	0	0	90
*Tenericutes*	88	0	0	0	0	0	0	0	85	3	0	0	0	0	0	0	86	2	0	0	88
*Spirochaetes*	33	29	20	0	0	0	0	0	69	10	2	1	0	0	0	0	60	22	0	0	82
*Negativicutes*	4	56	1	1	0	0	0	0	1	43	11	5	1	1	0	0	55	4	2	1	62
*Cytophagia*	26	9	15	2	2	0	0	0	6	38	9	1	0	0	0	0	48	6	0	0	54
*Deinococcusthermus*	2	25	7	3	4	0	0	0	26	13	1	1	0	0	0	0	41	0	0	0	41
*Sphingobacteriia*	4	10	15	4	1	0	0	0	30	3	0	1	0	0	0	0	33	1	0	0	34
*Fusobacteria*	27	4	0	0	0	0	0	0	29	2	0	0	0	0	0	0	30	1	0	0	31
*Erysipelotrichi*	19	4	0	1	0	0	0	0	10	8	4	1	1	0	0	0	18	6	0	0	24
*Chlamydiae*	14	6	1	0	0	0	0	0	20	1	0	0	0	0	0	0	21	0	0	0	21
*Thermotogae*	16	1	0	0	0	0	0	0	17	0	0	0	0	0	0	0	17	0	0	0	17
*Aquificae*	8	4	3	1	0	0	0	0	16	0	0	0	0	0	0	0	3	11	2	0	16
Bacteria	3	10	1	2	0	0	0	0	7	9	0	0	0	0	0	0	15	1	0	0	16
*Planctomycetes*	2	8	5	0	0	1	0	0	7	5	1	1	2	0	0	0	13	2	1	0	16
*Synergistetes*	1	13	2	0	0	0	0	0	11	4	1	0	0	0	0	0	16	0	0	0	16
*Chlorobi*	0	0	7	6	0	0	0	0	13	0	0	0	0	0	0	0	12	1	0	0	13
*Acidobacteriia*	0	0	1	7	4	0	0	0	11	1	0	0	0	0	0	0	0	8	4	0	12
*Verrucomicrobia*	2	5	3	0	0	0	0	0	4	3	2	1	0	0	0	0	10	0	0	0	10

a Taxonomic assignment is based on the annotation level of the genome in eggNOG 4.5 (38).

Importantly, all DedA homologs thought to have UndP flippase activity (7, 8) are in this subfamily, including the four E. coli DedAs that can singly support viability (9) (and therefore putatively have UndP flippase activity), the 2 Pseudomonas aeruginosa DedAs that can complement nonlethal phenotypes of a Δ2-DedA E. coli strain (20), and the 2 B. subtilis DedAs whose deletion affects MX2401 sensitivity (7). These data suggest that UndP flippase activity is the hallmark of the COG0586 DedA subfamily.

The conservation of key residues strengthens this hypothesis. The core DedA domain (1, 2), which likely forms a homodimer (2), consists of an α-helical bundle with 2 reentrant helices and 3 transmembrane helices (Fig. 2A), a conserved (Fig. S1) structure similar to that in other transporter families (2). In such transporters, the residues at the tips of the two at reentrant helices are invariably involved in substrate interactions. COG0586 DedAs contain conserved acidic residues (Fig. 2) at the tip of the first reentrant helix (YghB^Eco^ Asp51 and Glu39) (Fig. 2B and C) that likely bind protons (2), and conserved basic residues (Fig. 2B and C) at the tip of the second reentrant helix (YghB^Eco^ Arg130 and Arg136) that likely bind the negatively charged phosphate group of UndP (7). These residues have been shown to be essential in both E. coli (21) and B. subtilis (7) COG0586 proteins, and together they likely effect proton motive force (PMF)-driven flipping of UndP.

Genomic analyses have provided additional evidence for the UndP flippase activity of the COG0586 subfamily. Although 93% of COG0586 family proteins contain only the PF09335 (DedA) domain, fusions to PF00581 (rhodanese) and PF01569 (PAP2) exist. PAP2 domains include phosphatidic acid phosphatases, which may dephosphorylate UndP-P prior to flipping; this is an essential function usually performed by a separate protein (e.g., BacA). Rhodanese domains are sulfurtransferases; their connection to UndP is unclear. Finally, COG0586 DedA genes are less common in genomes that also encode a non-DedA UndP flippase (7, 8) (e.g., COG2035, which contains DUF368), whereas no such relationship exists between COG2035 and the other subfamilies (Fig. S2). Together, these data suggest that COG0586 DedAs are UndP flippases and raise the possibility that environmental or regulatory specialization is responsible for the genomic redundancy of COG0586 genes.

10.1128/mbio.00028-23.2 FIG S2 The proportion of genomes with a COG0586 family homolog is highest when no DUF368 family UndP flippases are present in the same genome, and the proportion decreases as the number of DUF368 members increases. No such pattern was observed for the other two subfamilies. Download FIG S2, TIF file, 9.9 MB. Copyright © 2023 Todor et al. 2023 Todor et al. https://creativecommons.org/licenses/by/4.0/ This content is distributed under the terms of the Creative Commons Attribution 4.0 International license.

### COG0398 DedA proteins are associated with aerobic metabolism.

The COG0398 DedA subfamily is the second largest family, accounting for ~27% of bacterial DedAs. Intriguingly, eukaryotic DedAs, such as TMEM41B, TMEM64, and VMP1, are most closely related to this bacterial subfamily (22).

COG0398 DedAs are unlikely to function as UndP flippases, because they lack the acidic residues at the tip of the first reentrant helix associated with PMF-driven transport (Fig. 2B and D) and are missing one or both of the conserved basic residues at the tip of the second reentrant helix putatively required for binding UndP (Fig. 2B and D) (7, 23). The sequence of the putative substrate binding region at the tip of the second reentrant helix is similar to that of the eukaryotic DedA homologs, which have been characterized to function as phospholipid scramblases (3, 4). If bacterial COG0398 genes are also phospholipid scramblases, they may function to maintain or vary the phospholipid asymmetry of the inner membrane (24).

COG0398 DedAs exhibit a striking phylogenetic distribution (Table 1), being excluded from many predominantly anaerobic phyla (e.g., *Bacteroidetes*, *Chlorobi*) and classes (e.g., *Bifidobacteriales*, *Propionibacteriales*, *Actinomycetales*). Most COG0398 subfamily DedAs (90%) only contain the PF09335 (DedA) domain. The most common domain fusions are with PF07992 and PF02852 domains (pyridine nucleotide-disulfide oxidoreductase and dimerization), which are found in glutathione-thioredoxin reductase, lipoamide dehydrogenase, and mercuric reductase. Lipoamide and glutathione are associated with aerobic metabolism, consistent with the observed distribution of COG0398 DedAs. COG0398 DedA proteins are essential in the alphaproteobacterial model organisms Caulobacter crescentus and Dinoroseobacter shibae (25). Further studies in these organisms may reveal their function and connection to aerobic metabolism.

### COG1238 subfamily DedA proteins are predominantly found in Gram-negative bacteria.

The COG1238 subfamily accounts for only ~14% of bacterial DedAs, and little is known about its function. A COG1238 homolog has been reported to play a role in indium resistance in *Rhodanobacter* sp. B2A1Ga4, but no mechanism was proposed (26).

COG1238 DedA subfamily proteins are also unlikely to function as UndP flippases; although the first reentrant helix exhibits conserved acidic residues suggestive of PMF-driven transport, the second reentrant helix is almost completely lacking in positive residues that could bind a phosphate (Fig. 2B and E). The absence of conserved positive residues in the putative substrate binding site in the second reentrant helix suggests that COG1238 subfamily DedAs may transport uncharged or even positively charged lipids. Almost all (99%) COG1238 subfamily proteins are single-domain proteins.

The COG1238 subfamily DedAs are predominantly found in *Proteobacteria*, *Bacteroidetes*, *Acidobacteriia*, and *Aquificae* (>50% of species in these clades), occasionally in *Cytophagia*, *Negativicutes*, *Planctomycetes*, *Flavobacteriia*, *Erysipelotrichi*, and *Spirochaetes* (10 to 25%), and rarely in other Gram-negative phyla or in Gram-positive phyla, such as *Actinobacteria*, *Bacilli*, and *Clostridia* (Table 1). It is unclear why this class is associated with such a specific subset of Gram-negative bacteria. One possibility is that COG1238 DedAs transfer specific lipids to the inner leaflet of the outer membrane (OM), potentially through a direct interaction with an AsmA family phospholipid bridge (27). Strikingly, this would mirror lipid transfers during autophagy, where eukaryotic DedA homologs transfer lipids to the autophagosome via ATG2, a protein homologous to bacterial AsmA proteins (28). COG1238 proteins are essential in several Pseudomonas species (25), and this opens the door to experimental characterization of their function.

### DedA proteins may be frequent antibiotic targets.

The lipopeptide antibiotic amphomycin, which inhibits UndP recycling, was key to identifying the role of the COG0586 DedA family (7, 8). We therefore asked whether additional antibiotics antagonize DedA activity. We reasoned that a modified DedA protein in the biosynthetic gene cluster (BGC) of such an antibiotic may provide immunity, as has been demonstrated for daptomycin (29) and speculated for others (29–31). We therefore searched the MIBiG database (32) of characterized BGCs for DedAs (PF09335) and identified 18 such clusters (Table S2). To more broadly ascertain DedA-containing BGCs, we next searched the antiSMASH database (33), which contains ~147,000 computationally predicted BGCs, for clusters containing smCOG1188 (homologous to DedA), and we identified 2,213 DedA-containing BGCs in diverse bacteria, including *Streptomyces*, *Bacillus*, and Pseudomonas (Table S2). Consistent with this broad distribution of putatively DedA-antagonizing antibiotics, oxydifficidin, a product of *Bacillus* species, was recently shown to kill Neisseria gonorrhoeae in a DedA-dependent manner (34). These observations suggest that antagonizing DedA function is a widespread antibiotic modality.

10.1128/mbio.00028-23.4 TABLE S2 DedA homologs identified in BGCs. Download Table S2, XLSX file, 0.3 MB. Copyright © 2023 Todor et al. 2023 Todor et al. https://creativecommons.org/licenses/by/4.0/ This content is distributed under the terms of the Creative Commons Attribution 4.0 International license.

### Summary and perspective.

Recent experimental work in bacteria and eukaryotes has uncovered a conserved lipid flippase or scramblase function for DedA family proteins (3, 4, 6–8, 22). Our bioinformatic and predicted structure analyses show that bacterial DedA proteins share a conserved core structure (Fig. 2C to E, Fig. S1) but have evolved into three families with distinct functional residues and phylogenetic profiles (Fig. 1). These families likely predate the last bacterial common ancestor. The COG0586 family is likely involved in UndP recycling, the COG0398 family is associated with aerobic metabolism, and the COG1238 family is associated with the Gram-negative OM.

Future experimental studies of DedA family proteins in bacteria will shed new light on the diversity, function, and trafficking of bacterial lipids. Understanding the substrate specificity, phenotypes, genetics, and regulation of the COG0398 DedA family can elucidate the link between aerobic metabolism and the membrane, while understanding the COG1238 DedA family can reveal novel aspects of the Gram-negative outer membrane. Additionally, the putative presence of many DedA-antagonizing antibiotics in the genomes of *Actinobacteria* and *Bacilli* may provide useful membrane-targeting antibiotics.

### Methods.

**(i) Identification of DedA homologs.** To identify DedA homologs, we used hmmer 3.3.2 to query the proteomes of ~6,000 representative bacterial genomes from the Progenomes1 (35) database with the PF09335.14 motif characteristic of DedA family proteins. This process identified and aligned ~17,000 DedA homologs (Table S1). We excluded ~600 proteins from poorly represented phyla and used the remaining set of 16,100 proteins for downstream analysis. The set of 16,100 proteins was annotated using eggNOG-mapper v2, and 15,993 of the sequences were successfully annotated (>99%).

**(ii) Alignment.** Because the PF09335.14 sequence does not include the entirety of the first reentrant helix, we realigned the 16,100 sequences using the structure-aware multiple-alignment program PROMALS3D (36).

**(iii) Tree construction.** We constructed a tree using the DedA domain (PF09335.14) of 986 randomly chosen DedA proteins, plus all DedA homologs in B. subtilis and E. coli (a total of 1,000 sequences). Briefly, we considered only the columns of the alignment in which at least 95% of sequences had an aligned residue and calculated distances using BLOSUM62. The tree was constructed using the UPGMA algorithm and colored by protein membership in previously computed COGs (eggNOG 5.0 [37, 38]).

**(iv) Structure prediction and analysis.** AlphaFold models were acquired from the AlphaFold database using the following identifiers: YghB^Eco^ (P0AA60), YdjZ^Eco^ (P76221), YqaA^Eco^ (P0ADR0), YkoX^Bsub^ (O34908), YqeD^Bsub^ (P54449), and BT3251 (Q8A2Q4). All models were aligned and modeled with PyMOL 2.5.4.
