# Supplementary figures and images for: Three Bacterial DedA Subfamilies with Distinct Functions and Phylogenetic Distribution
Source: mBio. 2023 Mar 1;14(2):e00028-23. doi: 10.1128/mbio.00028-23 (PMC10127716; doi:10.1128/mbio.00028-23)

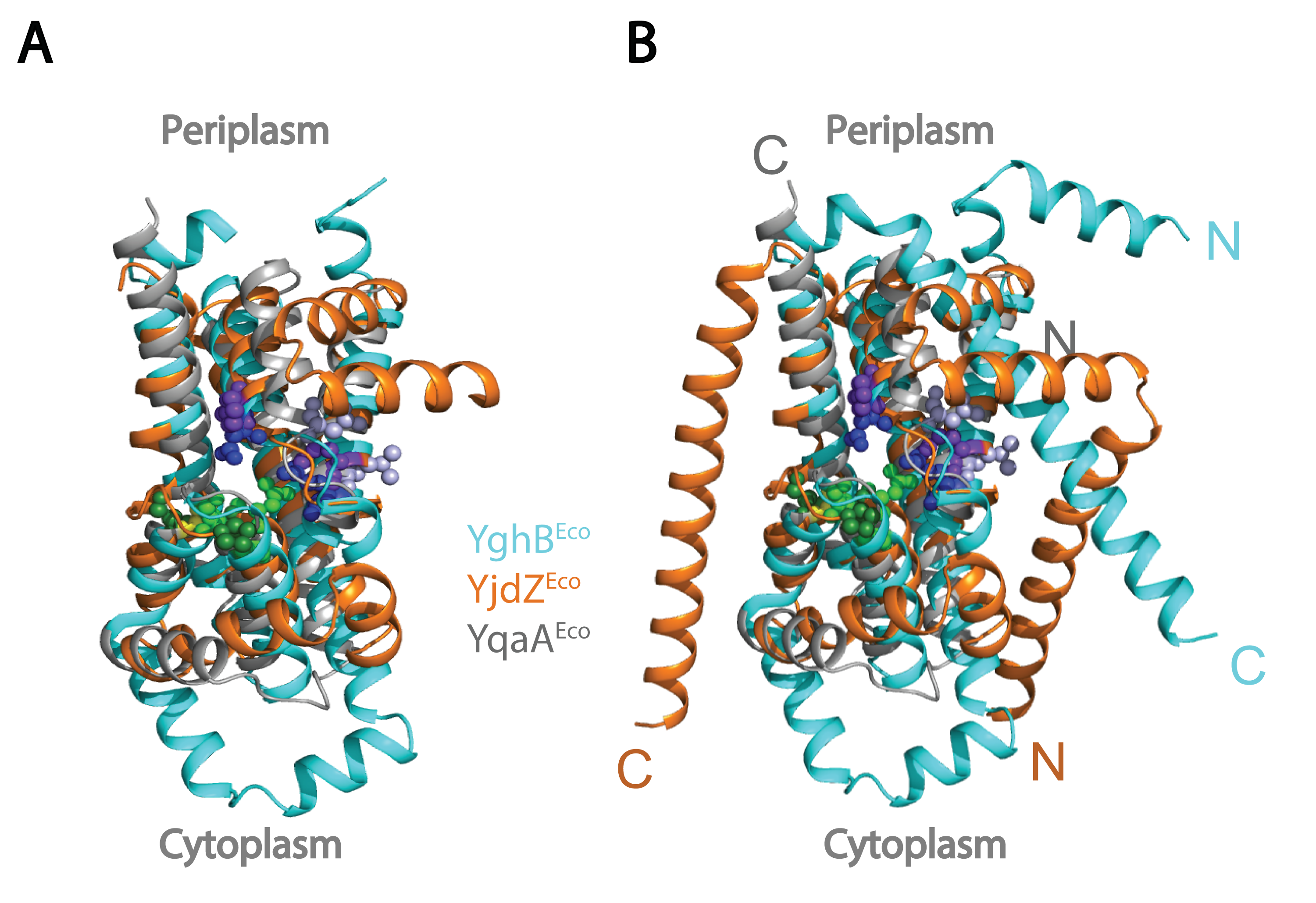

Supplement: FIG S1 [file mbio.00028-23-s0002.tif]

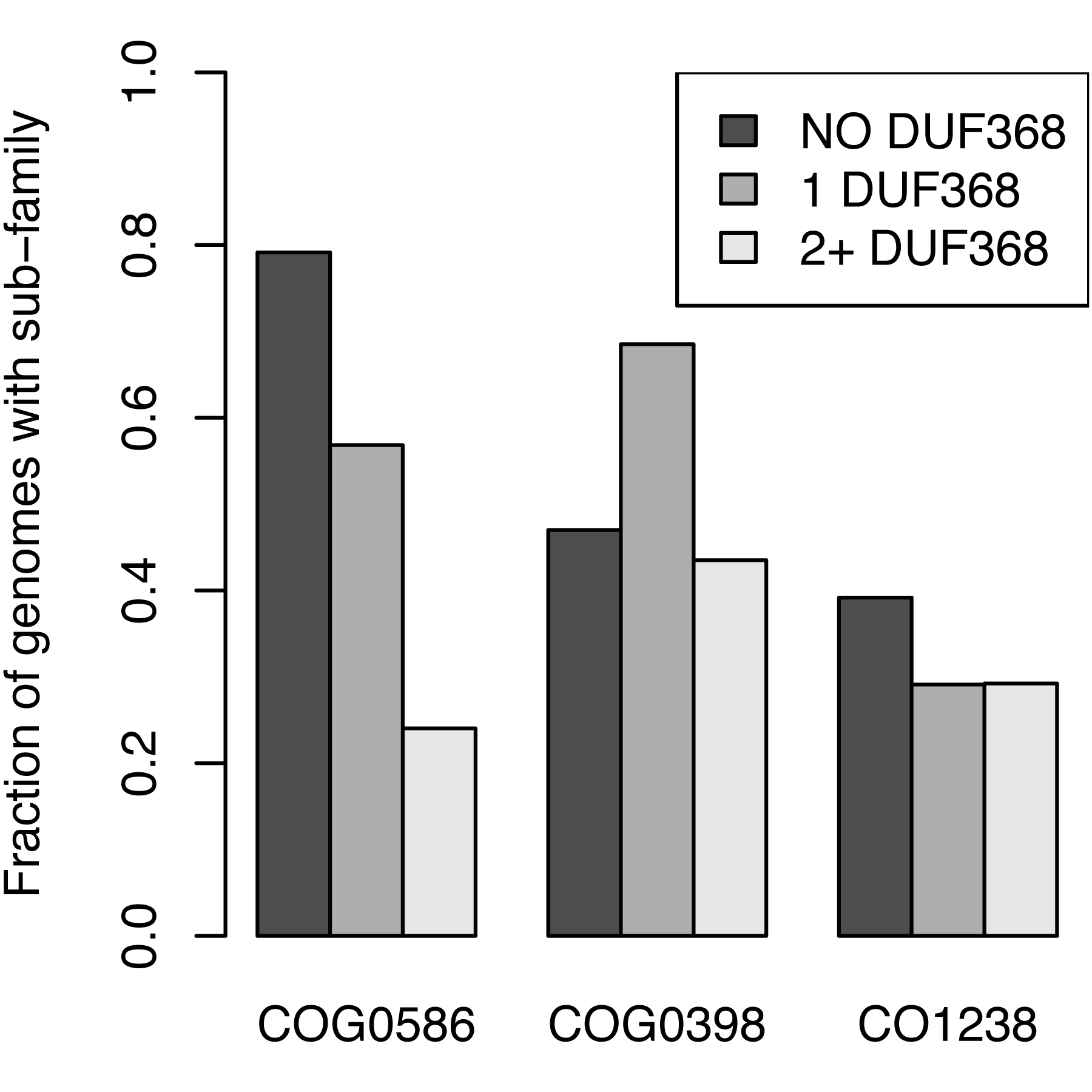

Supplement: FIG S2 [file mbio.00028-23-s0003.tif]
